# Protecting digital assets using an ontology based cyber situational awareness system

**DOI:** 10.3389/frai.2024.1394363

**Published:** 2025-01-09

**Authors:** Tariq Ammar Almoabady, Yasser Mohammad Alblawi, Ahmad Emad Albalawi, Majed M. Aborokbah, S. Manimurugan, Ahmed Aljuhani, Hussain Aldawood, P. Karthikeyan

**Affiliations:** ^1^Faculty of Computers and Information Technology, University of Tabuk, Tabuk, Saudi Arabia; ^2^NEOM, Tabuk, Saudi Arabia; ^3^RV University, Bengaluru, India

**Keywords:** anomaly detection, cyber situational awareness, structured threat information expression, isolation forest algorithm, auto encoder

## Abstract

**Introduction:**

Cyber situational awareness is critical for detecting and mitigating cybersecurity threats in real-time. This study introduces a comprehensive methodology that integrates the Isolation Forest and autoencoder algorithms, Structured Threat Information Expression (STIX) implementation, and ontology development to enhance cybersecurity threat detection and intelligence. The Isolation Forest algorithm excels in anomaly detection in high-dimensional datasets, while autoencoders provide nonlinear detection capabilities and adaptive feature learning. Together, they form a robust framework for proactive anomaly detection.

**Methods:**

The proposed methodology leverages the Isolation Forest for efficient anomaly identification and autoencoders for feature learning and nonlinear anomaly detection. Threat information was standardized using the STIX framework, facilitating structured and dynamic assessment of threat intelligence. Ontology development was employed to represent knowledge systematically and enable semantic correlation of threats. Feature mapping enriched datasets with contextual threat information.

**Results:**

The proposed dual-algorithm framework demonstrated superior performance, achieving 95% accuracy, a 99% F1 score, and a 94.60% recall rate. These results outperformed the benchmarks, highlighting the model’s effectiveness in proactive anomaly detection and cyber situational awareness enhancement.

**Discussion:**

The integration of STIX and ontology development within the proposed methodology significantly enhanced threat information standardization and semantic analysis. The dual-algorithm approach provided improved detection capabilities compared to traditional methods, underscoring its potential for scalable and effective cybersecurity applications. Future research could explore further optimization and real-world deployments to refine and validate the approach.

## 1 Introduction

The issue of cybersecurity is increasingly concerning in contemporary society, as information technology systems and networks are assumed to play a vital role as critical infrastructure for diverse sectors and institutions. The human user is often considered the weakest link in cybersecurity; thus, understanding human behavior is crucial relative to developing effective security products (Fghdhfgh, 2022). Situational awareness, defined as the cognitive process of understanding and interpreting environmental conditions and events, holds critical importance in decision-making, particularly in the context of ensuring accurate and optimal choices as well as averting incidents and mishaps that are attributable to inadvertent misjudgments and mistakes committed by human individuals. Endsley defined situational awareness as perceiving and comprehending environmental elements and projecting their status soon (Avdeenko and Makarova, 2018). In the cybersecurity context, situational awareness is important for cybersecurity and requires the involvement of human analysts in data fusion and decision-making processes (Alosaimi and Almutairi, 2023). In this context, the term “cyber situational awareness” refers to the organization’s ability to comprehensively understand its cybersecurity landscape, including its current security posture, potential vulnerabilities, and active threats. By enhancing situational awareness, organizations can better anticipate and mitigate cyber risks, thereby protecting their digital assets and maintaining business continuity (Munir et al., 2021; Friedberg et al., 2015).

The research problem in the domain of cyber situational awareness pertains to the pressing necessity of formulating and materializing a sophisticated and automated system for detecting and identifying intrusions that are deemed advanced and persistent in nature. Currently, these types of attacks are primarily detected post facto through forensic analysis by experts, which is an art rather than a science. The goal is to automate the analysis by grounding it in formal logic, ontologies, generative graph grammars, and reasoning under uncertainty. This approach would enable reasoning over attack vectors, attack targets, and knowledge of the system elements, making cyber defense more adaptive (Alyami and Almutairi, 2022; Mehraj et al., 2023; Kim et al., 2023).

### 1.1 Motivations

1. Continuous risk assessment and management are of utmost importance due to the increasing number of malicious actions and the need to maintain risk within acceptable limits. This study attempts to tackle the challenge of managing risk in real-time within a unified secure environment encompassing physical and logical elements (Välja et al., 2020).
2. Through the utilization of sensor-based systems and a current inventory of vulnerabilities, it becomes feasible to identify anomalies and acquire a comprehensive understanding of the level of risk associated with the system.
3. This project was driven by the need to construct a framework that can dynamically evaluate system risk, establish security metrics, and assess both short-term and long-term ramifications.
4. Real-time management is indispensable for evaluating the impact of incidents on the system as they are identified (Saeed et al., 2023; Ramzan et al., 2021).

The primary objective of this study was to detect and handle attacks at an early stage. We propose a framework that includes an ontology and models to collect data, obtain security metrics, correlate cyber incidents, examine sources and sensors, issue alerts, analyze issues, and facilitate decision-making. The use of ontologies is applicable in the risk management domain. The challenge of reasoning and modeling the residual risk of a system is addressed by considering anomalies, asset, and threat information.

The remainder of this paper is organized as follows. Section 2 discusses related works. The proposed system is presented in Section 3. The results and discussion are described in section 4. Finally, we conclude the paper and outline future research directions in section 5.

## 2 Related works

Keshavarzi et al. proposed a framework to represent knowledge about digital extortion attacks using an ontology. The Rantology framework focuses primarily on ransomware attacks and leverages logic encoded in the ontology to assess the maliciousness of programs based on various factors like called API (Keshavarzi and Ghaffary, 2023). Khaldian et al. presented a highly effective approach for real-time anomaly detection and classification in synchrophasor data using Isolation Forest and K-means algorithms. Their method achieved notable success in identifying both event-related and data-quality anomalies with impressive recall rates. This area is crucial for smart grid reliability and security because anomalous synchrophasor data can significantly impact grid operations, control systems, and situational awareness. The fusion of Isolation Forest and K-Means leverages their complementary strengths while mitigating individual limitations (Khaledian et al., 2021).

Riesco et al. addressed the critical objective of organizations to maintain risks at acceptable levels over time given their constant exposure to security threats. It highlights emerging challenges such as undetected tactics, asset decentralization, IoT vulnerabilities, and false positives. Existing risk management frameworks often lack integration and automation with near real-time cybersecurity threat intelligence (CTI). This paper proposes an integrated architecture that utilizes the web ontology language (OWL), semantic reasoning, and Semantic Web Rule Language (SWRL) to establish a Dynamic Risk Assessment and Management (DRA/DRM) framework across operational, tactical, and strategic levels (Riesco and Villagrá, 2019). Sánchez-Zas et al. (2023) proposed a novel approach for real-time risk management and cyber-situation awareness using an ontology-based framework. Their method leverages ontologies to model cyber threats, assets, vulnerabilities, and relationships among them, enabling dynamic risk assessment and real-time visualization of cyber-situations. This study addresses the critical need for efficient and comprehensive cyber defense strategies in increasingly complex environments.

Cauteruccio et al. (2021) discussed the complexities of anomaly investigation in Multiple IoT (MIoT) environments, where interconnected networks of people and devices interact. Recognizing the need for more research in this field, the authors proposed a methodological framework to guide future explorations. They introduced two key concepts: the forward problem and the inverse problem, which allow researchers to systematically analyze how anomalies are influenced by factors like distances between nodes, overall network size, and centrality measures of affected nodes. The proposed framework was then applied to a real-world smart city scenario, demonstrating its potential in leveraging sensor and social network data to optimize smart lighting and enhance citizen safety.

A cybersecurity system for managing situational awareness in critical infrastructures was created by Graf et al. (2016). The created techniques offer an automated decision support system and assist in resolving real-world situational awareness issues, such choosing whether or not to sound an alert. Friedberg et al. (2015) proposed a novel approach called AECID (Automatic Event Correlation for Incident Detection) is proposed, which addresses the limitations of common intrusion and anomaly detection mechanisms while also supporting privacy-aware information sharing for cyber situational awareness. Tan et al. (2021) proposed a novel approach to secure and enhance situational awareness in an Artificial Internet of Things (AIoT) environments using HoneyNets. Their method leverages strategically placed decoy devices to attract and capture cyberattacks, thereby enabling threat detection and analysis to improve overall system resilience. This study addresses the growing concerns about security vulnerabilities in AIoT, where interconnected devices and intelligent functionalities require robust protection. Jirsik and Celeda (2020) proposed a framework for cyber-situation awareness using IP flow monitoring. The proposed method employs novel flow monitoring and analysis techniques to detect and understand anomalies in network traffic, thereby enhancing the understanding of the cyber landscape and facilitating proactive responses. This study addresses the crucial need for effective cybersecurity solutions in increasingly complex and interconnected networks.

Nota and Petraglia (2024) focused on the critical mission of preserving cultural heritage through the use of technological advancements for monitoring and conservation purposes. They highlight the significance of integrating theoretical insights into practical implementations to ensure effective protection of these invaluable assets. Recent technological progress has facilitated the development of advanced monitoring and control systems that provide accurate and timely insights into the condition of heritage structures. By adopting a situational awareness model as the basis, this study proposes a framework for crafting and deploying cyber-physical systems to bolster conservation endeavors. Papadopoulos et al. (2024) proposed an advanced framework designed to protect IT resources from attackers. Attackers can be from outside or inside the infrastructure. The developed framework supports a response coordination system and aids decision-making by offering mitigation strategies and sharing information with relevant authorities and the publisher the developed model may not support online cloud service resources. Park et al. (2019) work can be seen as contributing to this area by proposing a framework that incorporates situational awareness to enable more comprehensive risk measurements. This study explores various metrics to quantify the risks associated with security vulnerabilities in IoT devices. These metrics consider factors like exploitability, likelihood of occurrence, and potential impact on privacy and safety. Table 1 lists the existing related works.

**Table 1:** Summary of existing related works.

Authors and years of study	Techniques	Methodology	Merits	Demerits
Keshavarzi and Ghaffary (2023)	An ontology-based framework (Rantology) to represent knowledge about digital extortion attacks, primarily focusing on ransomware.	Develop an ontology-based framework (Rantology) to represent ransomware attack knowledge and assess program maliciousness using logic and API factors.	Provides a structured approach to ransomware assessment, enhancing understanding and response to digital extortion.	Limited to ransomware attacks, may not be generalizable to other types of digital extortion.
Khaledian et al. (2021)	Real-time anomaly detection and classification in synchrophasor data using Isolation Forest and K-Means.	Combine Isolation Forest and K-Means for real-time anomaly detection in synchrophasor data, focusing on event-related and data-quality anomalies.	Effective real-time anomaly detection, crucial for smart grid security and operational reliability.	It may require significant computational resources for real-time processing.
Riesco and Villagrá (2019)	Integrated architecture using Web Ontology Language (OWL), semantic reasoning, and Semantic Web Rule Language (SWRL) for Dynamic Risk Assessment and Management (DRA/DRM).	Utilize OWL, semantic reasoning, and SWRL to create an integrated DRA/DRM framework for real-time cybersecurity threat intelligence integration and automation.	Integrates real-time threat intelligence with risk management, improving response to emerging cybersecurity challenges.	Complexity in integrating and automating real-time threat intelligence with existing systems.
Sánchez-Zas et al. (2023)	An ontology-based framework for real-time risk management and cyber-situational awareness.	Leverage ontologies to dynamically assess risk and visualize cybersecurity awareness by modelling threats, assets, vulnerabilities, and their relationships.	Provides dynamic risk assessment and real-time situational awareness, enhancing cybersecurity defense.	Ontology-based models are complex and require extensive updates to remain relevant.
Cauteruccio et al. (2021)	Methodological framework for anomaly investigation in Multiple IoT (MIoT) environments.	Proposed a methodological framework to study the influences of MIoT anomalies, applying it to a smart city scenario to optimize smart lighting and enhance safety.	Systematic analysis of MIoT anomalies, applicable to real-world scenarios to optimize safety and functionality.	Frameworks may be challenging to implement in diverse and heterogeneous MIoT environments with varying factors.
Graf et al. (2016)	System for situational awareness in critical infrastructure within cybersecurity.	Implement a system incorporating complex rules and automated decision support to enhance situational awareness in critical infrastructure cybersecurity.	Automates decision support in cybersecurity, improving situational awareness and incident response.	It may not cover all aspects of situational awareness, requiring supplementary methods for comprehensive coverage.
Friedberg et al. (2015)	AECID (Automatic Event Correlation for Incident Detection).	Develop an AECID for incident detection and privacy-aware information sharing to address common intrusion and anomaly detection limitations.	The proposed method addresses the limitations of traditional detection methods and enhances privacy-aware information sharing.	Potential privacy concerns in information sharing despite a privacy-aware design.
Tan et al. (2021)	Securing AIoT environments using HoneyNets.	Use HoneyNets to detect and analyze cyberattacks in AIoT environments, improving system resilience through strategically placed decoy devices.	Enhances AIoT security and resilience by effectively detecting and analyzing threats.	Dependence on decoy devices, which may be detected by advanced attackers.
Jirsik and Celeda (2020)	Cyber situation awareness using IP flow monitoring.	Employ IP flow monitoring and novel analysis methods to enhance cyber situation awareness by detecting and understanding network traffic anomalies.	Provides proactive understanding and response to network anomalies.	Continuous updates and adjustments may be required to handle evolving network threats.
Nota and Petraglia (2024)	Technological advancements for monitoring and conserving cultural heritage.	Integrate technology advancements in monitoring and control systems to protect cultural heritage, using a situational awareness model for cyber-physical systems.	Applies advanced technology to the preservation of cultural heritage, ensuring timely and accurate monitoring.	High initial setup and maintenance costs for advanced monitoring systems.
Papadopoulos et al. (2024)	Framework to protect IT resources from attackers.	Develop a framework supporting IT resource protection and response coordination, providing mitigation strategies, and sharing information, excluding cloud services.	Enhance IT resource protection through coordinated response and mitigation strategies.	Excludes online cloud services, thereby limiting its applicability in cloud-based environments.
Javadnejad et al. (2024)	AI-based tools and Zero Trust Architecture (ZTA) for combating attacks.	AI-based tools are software applications that use artificial intelligence (AI) to simulate human intelligence	This section provides a detailed analysis of ransomware malware risks and highlights the economic impact of different malware types.	Heavily rely on the quality and comprehensiveness of the Advisen cyber loss dataset, which may have limitations

### 2.1 Research gap

Despite advancements in cybersecurity situational awareness (CSA), significant gaps remain. Existing methods primarily rely on post-incident forensic analysis and predefined rule-based systems, which are insufficient for detecting advanced and persistent threats in real time. Current machine learning approaches require extensive feature engineering and lack dynamic adaptation to new threats. Additionally, existing risk management frameworks struggle with integration and automation, failing to provide real-time risk assessment and decision support. Addressing these gaps necessitates developing innovative, automated systems that leverage formal logic, ontologies, and advanced machine learning techniques to detect threats in real time, dynamically adapt to new threats, and integrate comprehensive risk management for enhanced cybersecurity.

## 3 Intelligent cyber situational awareness system

The proposed system utilizes advanced technologies, likely including artificial intelligence, to monitor the cyber landscape in real-time. By employing intelligent algorithms, the system can detect and respond to potential threats promptly, thereby enhancing the organization’s ability to protect its digital assets effectively. The focus is on maintaining comprehensive situational awareness of the cyber environment to proactively mitigate risks and ensure the security of critical digital assets. IT infrastructure encompasses a diverse array of resources, including server systems, end users, intermediary devices, such as routers and switches, and cloud services software. Collectively, these resources form the backbone of operations within an organization. Our proposed system aims to effectively manage access to these resources. Users are granted access to these resources exclusively through our system; attempting to bypass this system would result in denial of access to the resources in the IT infrastructure. To enhance security in this infrastructure, we developed a sophisticated deep learning model.

**Figure 1:**
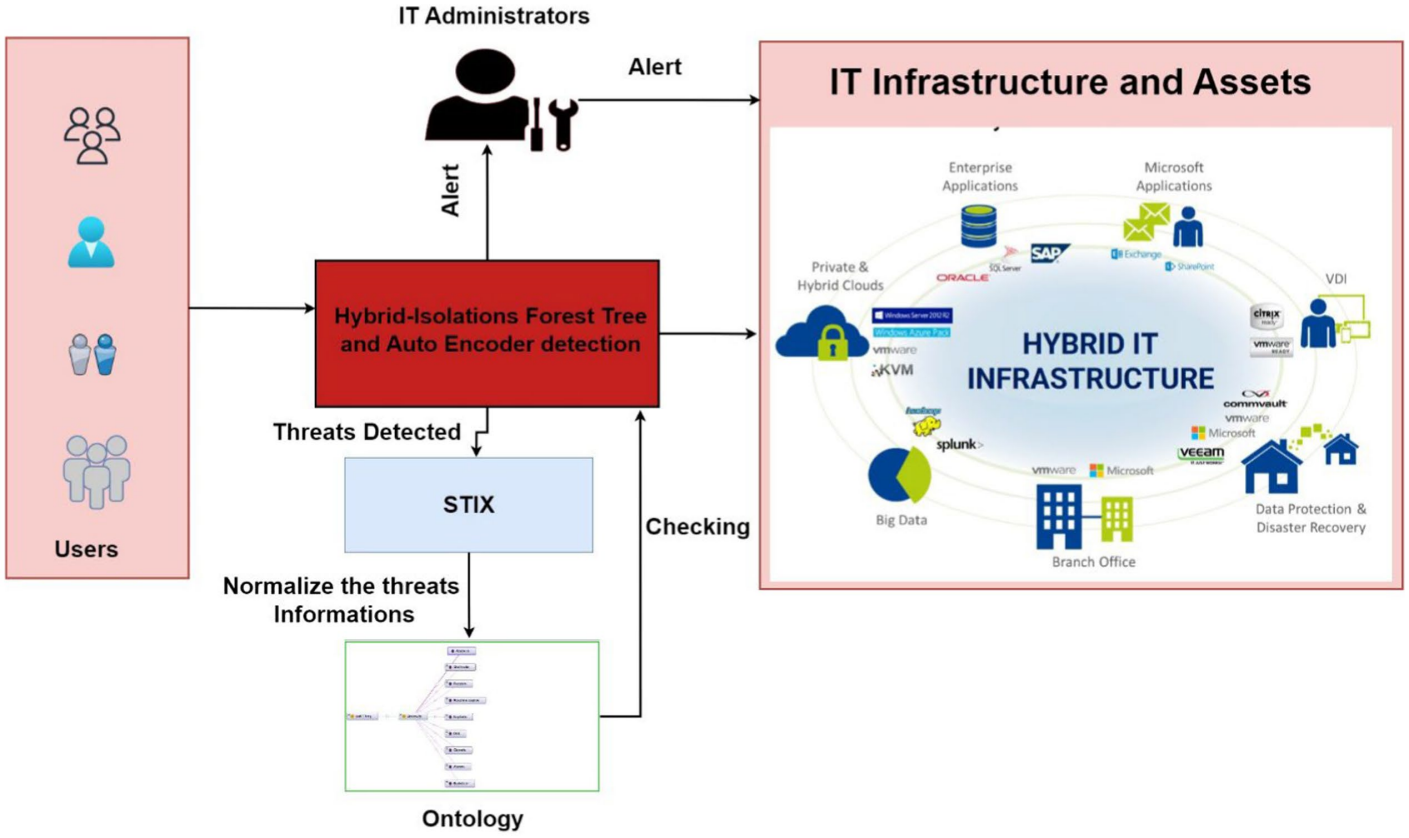
Overview of the intelligent based cyber situational awareness system.

Figure 1 presents an overview of the proposed system. The proposed model incorporates both isolation forest trees and an autoencoder, enabling it to identify and flag potential threats related to resource access. When a threat is identified, it undergoes a structured process for handling. Initially, it is relayed to the STIX protocol, which standardizes the threat information for further analysis. The information is then passed to an ontology-based system. Here, an ontology rule is generated based on threat information. This rule serves as a guideline for our deep learning model, facilitating the validation of user actions and the detection of potentially harmful activities that compromise the integrity of IT resources. Should the model detect suspicious or harmful activities initiated by a user, an alert mechanism is triggered to notify IT infrastructure managers promptly. This proactive approach enables swift intervention to safeguard resources and maintain the security of IT infrastructure.

We used multi-factor authentication (MFA), which strengthens security by requiring users to verify their identity using two or more different methods: something they know (password or PIN), something they have (smartphone or hardware token), and something they are (biometric data like fingerprints or facial recognition). The proposed MFA provides a secure authentication model to access organizational resources.

The proposed methodology uses the Isolation Forest algorithm and an autoencoder-based hybrid algorithm. This algorithm was selected for its well-documented efficacy in identifying anomalies in high-dimensional datasets, which is a critical requirement in the ever-evolving and dynamic cybersecurity landscape. The robust nature of this unsupervised learning algorithm is the cornerstone of the proposed anomaly detection framework, providing a solid foundation for the subsequent steps. The second step in the proposed methodology is the training process, where the algorithm is exposed to preprocessed and semantically enriched datasets. The careful preprocessing included data cleaning, feature selection, normalization/standardization, and noise removal, ensuring the removal of noise and irrelevant features, and setting the stage for the algorithm to discern meaningful patterns. Semantic enrichment enhances the algorithm’s contextual understanding of the cybersecurity domain, which is a crucial aspect for effective anomaly detection. This rigorous training regimen optimizes the algorithm’s capabilities, thereby emphasizing efficiency and precision, and makes it well-equipped to isolate anomalies in real-time cyber threat scenarios.

### 3.1 STIX integration

The third step introduces the integration of Structured Threat Information eXpression (STIX), which is a pivotal enhancement for our system’s threat intelligence capabilities. STIX serves as a standardized language, facilitating the expression of detailed threat information in a structured format. Simultaneously, feature mapping strategically links essential features, such as src_IP and dest_IP, to STIX indicators. This strategic linkage enhances the semantic understanding of network traffic and enriches the dataset with contextual threat information. The alignment of features with specific STIX patterns is instrumental in creating a more nuanced representation of cyber threats, which contributes to the overall sophistication of the proposed system.

### 3.2 Feature mapping

Feature Mapping for the UNSW-NB15 dataset involves transforming raw network traffic data into structured and enriched format to improve threat detection. This process includes collecting data, preprocessing data to handle missing values and normalizing features, and extracting relevant characteristics, such as protocol, service, and network flow specifics. These features are then semantically enriched using threat intelligence databases and ontologies by adding context such as geographical information and usage patterns. The enriched features are organized into a structured feature map using graph-based techniques, highlighting the relationships between different data points. This comprehensive feature map is integrated into anomaly detection models such as Isolation Forests and autoencoders, to enhance their ability to identify subtle patterns and anomalies. The results demonstrate improved contextual understanding, higher detection accuracy, and better scalability for real-time threat detection.

### 3.3 Ontology creation

The fourth step involves the creation of an ontology—a structured representation of knowledge within the cybersecurity domain. This foundational structure provides a conceptual framework for organizing and categorizing various entities, including but not limited to IP addresses, network protocols, attack categories, and security events. The ontology serves as a fundamental structure, allowing systematic categorization and correlation of different elements. This enhances the system’s ability to interpret and contextualize cyber threat data, setting the stage for advanced analysis and correlation methodologies. The creation of the ontology is a strategic move toward fostering clarity, consistency, and depth in our understanding of the cybersecurity landscape, posture (Algorithm 1).

#### Algorithm 1 Hybrid anomaly detection

**Table:** 

1: **Input:** Dataset: *cyber activity data* 2: **Preprocessing** 3: cyber activity data ←normalizeFeatures (*cyber activity data*) 4: **Isolation Forest Training:** 5: **function** Train Isolation Forest (*cyber activity data*) 6:Initialize an empty forest 7:**for** each tree in the forest **do** 8:Select a random subset of features from *cyber activity data* 9:Build an isolation tree using the selected features 10:Add the tree to the forest 11:**end for** 12: **end function** 13:**Autoencoder Training:** 14: **function** Train Autoencoder (*cyber activity data*) 15:Split the dataset into training and validation sets 16:Design and train an autoencoder neural network 17:**return** trained autoencoder 18: **end function** 19: **Anomaly Detection:** 20: **function** Detect Anomalies (*cyber activity data, trained autoencoder*) 21:**for** each *data point* in *cyber activity data* **do** 22:*isolation forest score*← **calculate isolation forest score** (*data point*) 23:Use the trained autoencoder to reconstruct *data point* 24:*hybrid anomaly score* ←**combine scores** (*isolation forest scrore, reconstruction* *error*) 25:**end for** 26:**return** hybrid anomaly scores 27: **end function** 28: **Thresholding:** 29: **function** Thresholding (*hybrid anomaly scores*) 30:Determine a threshold based on *hybrid anomaly scores* 31:**return** threshold 32: **end function** 33: **Anomaly Detection with Threshold:** 34: **function**Detect Anomalies With Threshold (*cyber activity data*, *trained autoencoder, threshold*) 35:*Hybrid anomaly scores*←**Detect Anomalies** (*cyber activity data, trained* *autoencoder*) 36:*Anomalies*← **filter data points above threshold** (*hybrid anomaly scores,* *threshold*) 37:**return** Anomalies 38: **end function**

X is the input data, f_IF_ (X) is the output of the Isolation Forest algorithm, which assigns an anomaly score to each data point. f_AE_ (X) is the output of the autoencoder, which reconstructs the input data. *λ* as the weight parameter to balance the contributions of the Isolation Forest and autoencoder. The input can X can be written the Equation 1.


(1) $$X\;\left(\begin{array}{ccc} X11 & X12\cdots & X13\\ X21 & X22 & X23\\ Xn1 & Xn2 & Xnm \end{array}\right)$$

The hybrid approach combines the anomaly scores from the Isolation Forest with the reconstruction errors from the autoencoder is computed the Equation 2.


(2) $$f\;\mathrm{Hybrid}\;(\;\left(X\right)=\lambda \cdot f\;\mathrm{IF}\;(\;\left(X\right)+\left(1-\lambda \right)\cdot f\;AE\;\left(X\right)$$

Where f_Hybrid_ (X) is the combined anomaly score for each data point. *λ* is a hyperparameter that determines the weight given to the Isolation Forest score versus the autoencoder reconstruction error. It can be tuned through cross-validation. The final step is to set a threshold on f_Hybrid_ (X) to classify data points as normal or anomalous. Data points with anomaly scores above the threshold are considered anomalies. The Isolation Forest algorithm begins by randomly selecting subsets of data points and constructing isolation trees. This process involves recursively partitioning the data space using random feature selections and split values until certain termination conditions, such as reaching the maximum tree depth or having only one data point in a subset, are met. Mathematically, this can be represented by the Partition function. Partition (X_t_, ℎ), which partitions the subset X_t_ with a maximum depth ℎ.

After constructing the isolation trees, the algorithm calculates the average path length from the root of each tree to every data point. For a given data point x_i_, the average path length E[ℎ(x_i_)] is computed as the average of the path lengths across all trees, denoted by the function in Equation 3.


(3) $$E(h\left(x_{i}\right)=\frac{1}{T}\;\overset{T}{\underset{t=1}{\sum }}h_{t}\;\left(\;x_{i}\right)$$

Where h_t_ (x_i_) is the path length from the root to x_i_ in tree t. Next, the average path lengths are normalized using the expected average path length c(n) for a sample of n points. The function c(n) is calculated using Equation 4.


(4) $$c\left(n\right)=2.H\;\left(n-1\right)-\frac{2\left(n-1\right)}{n}$$

The H(i) is computed using Equation 5.


(5) $$H\left(i\right)=\overset{i}{\underset{j=1}{\sum }}\frac{1}{j}$$

The normalized anomaly score is computed using Equation 6.


(6) $$S\left(x_{i}\right)\;=2\frac{E(h\left(x_{i\;}\right)}{c\left(n\right)}$$

### 3.4 Semantic threat intelligence integration (STIX)

**Figure 2:**
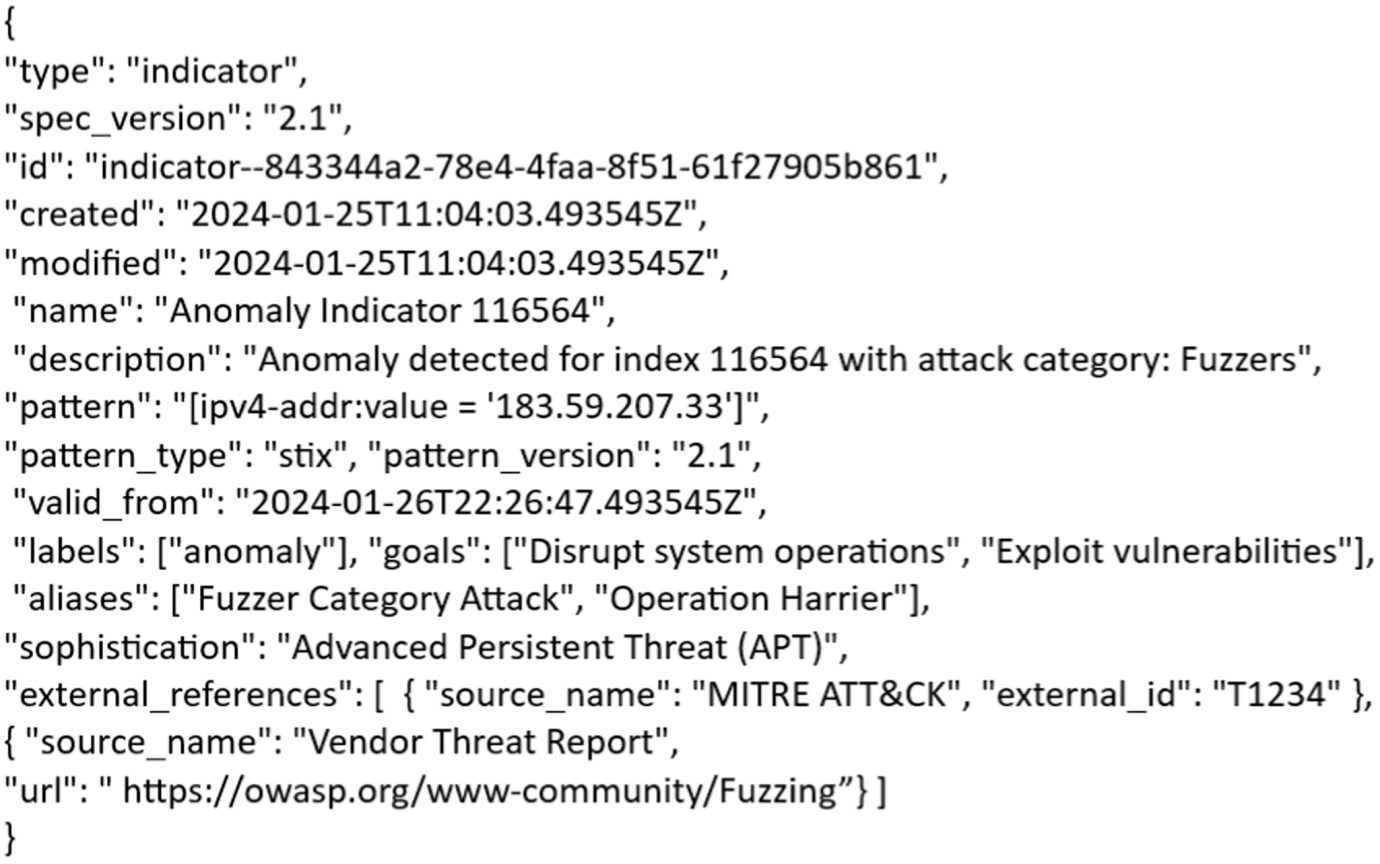
STIX indicator and its values.

The integration of Structured Threat Information eXpression (STIX) into the analysis framework marks a significant step toward enhancing the cyber threat intelligence capabilities of the system. STIX provides a standardized and interoperable language to express detailed threat information. By adopting STIX, the proposed framework gains the ability to represent cyber threat intelligence in a structured and machine-readable format, thereby ensuring consistency and facilitating seamless communication across different security tools and platforms. As part of the STIX implementation, a crucial aspect involves feature mapping, where the newly introduced src_IP and dest_IP features are systematically mapped to STIX indicators. This process enhances the semantic understanding of network traffic. The mapping involves associating features with specific STIX patterns representing indicators of anomalous or malicious activities. This linkage enables the assimilation of contextual threat information into the dataset, thereby creating a more enriched and nuanced representation of cyber threats. Figure 2 shows the STIX indicators and their values.

In STIX, Goals refer to the objectives or intended outcomes of a threat actor’s actions. This concept helps understand what the threat actor achieves with a particular attack or threat. The protocol allows the inclusion of this information to provide a context for the use of certain indicators or tactics. By analyzing the goals, organizations can better assess the threat’s potential impact, prioritize their responses, and develop more effective countermeasures.

Sophistication in STIX is related to the complexity and technical expertise required to execute a threat. STIX provides mechanisms to describe the level of technical skill or complexity involved in a threat actor’s methods. This includes details about the tools, techniques, and procedures (TTPs) used, and any advanced or novel techniques. Understanding such sophistication helps identify and address high-risk threats that require more advanced detection and defensive measures.

Aliases in STIX refer to various names or identifiers that may be used to describe the same threat or attack. This includes different terminologies, labels, or nicknames that different sources or organizations may use. STIX allows for the inclusion of these aliases to ensure that threat information is comprehensive and universally understandable, even if different sources use different terms to describe the same threat.

External References in STIX include additional sources of information that are not part of the immediate threat data but provide valuable context or validation. These references can include links to related reports, documents, or other data sources that support or enrich the threat information. By incorporating external references, STIX enhances the reliability and depth of threat analysis, ensuring that the threat data are well-rounded and corroborated by additional evidence or research.

### 3.5 Indicator description

The STIX framework represents a threat. For example, consider an STIX indicator related to an anomaly spotted in system traffic. The “Type” field specifies that the JSON object embodies a STIX indicator, which serves as a structured depiction of potential threats in cyber threat intelligence. The “Spec version” field is used to determine the version of the STIX, which is version 2.1 in this case.

- Id indicator (***“id”: “indicator--843344a2-78e4-4faa-8f51-61f27905b861”***) is used Unique identifier for the STIX indicator.
- ***“created”: “2024–01-25 T11:04:03.493545Z”:*** Indicates the date and time when the indicator was created, provided in ISO 8601 format with a time zone offset.
- ***“modified”: “2024–01-25 T11:04:03.493545Z”:*** Specifies the date and time when the indicator was last modified, also in ISO 8601 format.
- ***“name”: “Anomaly Indicator 116,564”:*** Provides a descriptive name for the indicator, helping to identify and categorize it.
- ***“description”: “Anomaly detected for index 116,564 with attack category: Fuzzers”:*** Offers a detailed description of the anomaly, providing information about the detected event and its association with the attack category “Fuzzers.”
- ***“pattern”: “[ipv4-addr:value = ‘78.213.93.129′]”:*** Represents the STIX pattern that defines the observable associated with the indicator. In this case, it is looking for an IPv4 address with the specific value ‘78.213.93.129′.
- ***“pattern_type”: “stix”:*** Specifies the type of pattern being used, indicating that it follows the STIX pattern syntax.
- ***“pattern_version”: “2.1”:*** Indicates the version of the STIX pattern language being used.
- ***“valid_from”: “2024–01-26 T22:26:47.493545Z”:*** Specifies the date and time from which the indicator is considered valid, in ISO 8601 format.
- ***“labels”: [“anomaly”]:*** Provides labels or tags associated with the indicator. In this case, it is labeled as an “anomaly.”
- MITRE ATT&CK (external_id: T1234): This refers to a specific technique in the MITRE ATT&CK framework, which is a globally accessible knowledge base of adversary tactics and techniques based on real-world observations. The external ID, T1234, links to the detailed description of a specific attack technique in the framework.

### 3.6 Ontology development

#### 3.6.1 Ontology creation

The creation of an ontology within the project is a fundamental aspect that significantly contributes to the system’s cyber threat intelligence capabilities. An ontology, in the cybersecurity context, serves as a formal and explicit representation of knowledge about entities and their relationships in the domain. This structured representation provides a conceptual framework to organize and categorize information relevant to cyber threats. Entities may include but are not limited to IP addresses, network protocols, attack categories, and security events. By establishing an ontology, the project establishes a shared understanding of the key concepts in the cybersecurity domain, thereby fostering clarity and consistency in the representation of knowledge.

#### 3.6.2 SPARQL integration

As part of the ontology creation, the project incorporates SPARQL (SPARQL Protocol and RDF Query Language), a query language and protocol used to retrieve and manipulate data stored in Resource Description Framework (RDF) format. SPARQL plays a crucial role in checking the presence and retrieval of threat information within the ontology. The queries in SPARQL enable the system to verify whether specific threat-related entities or relationships have been successfully saved in the ontology. This integration enhances the ontology’s utility by providing a mechanism to assess the presence of threat intelligence data dynamically, which ensures the system’s responsiveness to evolving cyber threats.

#### 3.6.3 Semantic correlation

The role of the ontology extends beyond mere organization; it plays a pivotal role in semantic correlation, a sophisticated method of relating information based on its meaning and context. By capturing the semantic context of cyber threats, the ontology becomes a critical component in deciphering the intricacies of the cybersecurity landscape.

**Figure 3:**
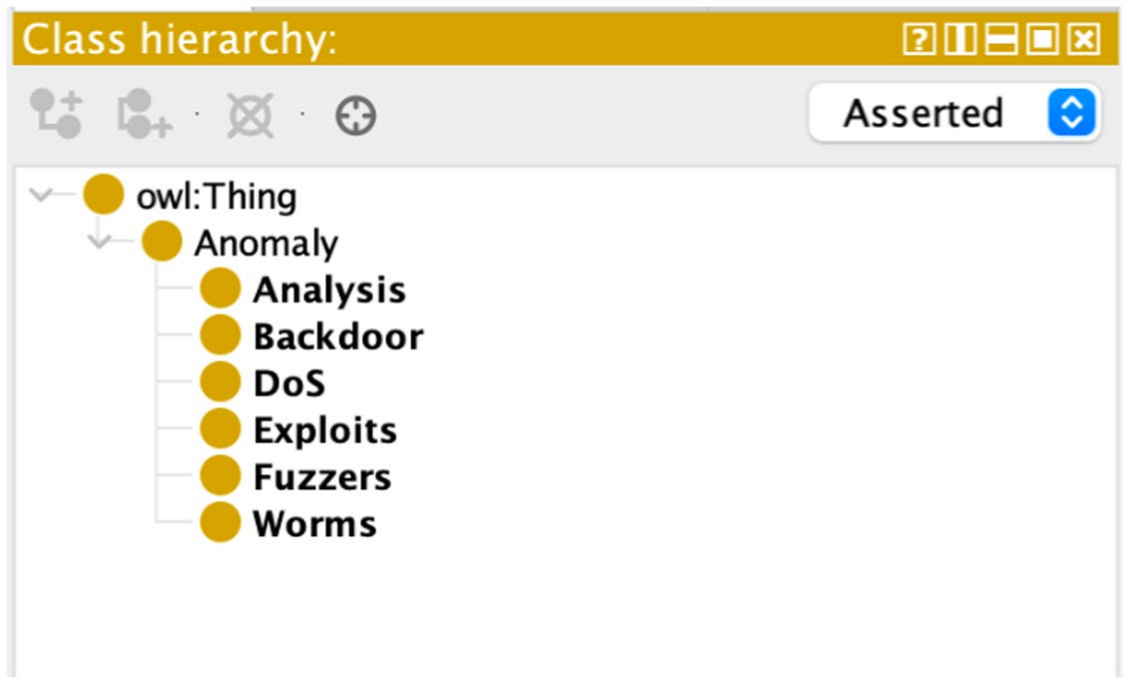
Anomaly hierarchy.

**Figure 4:**
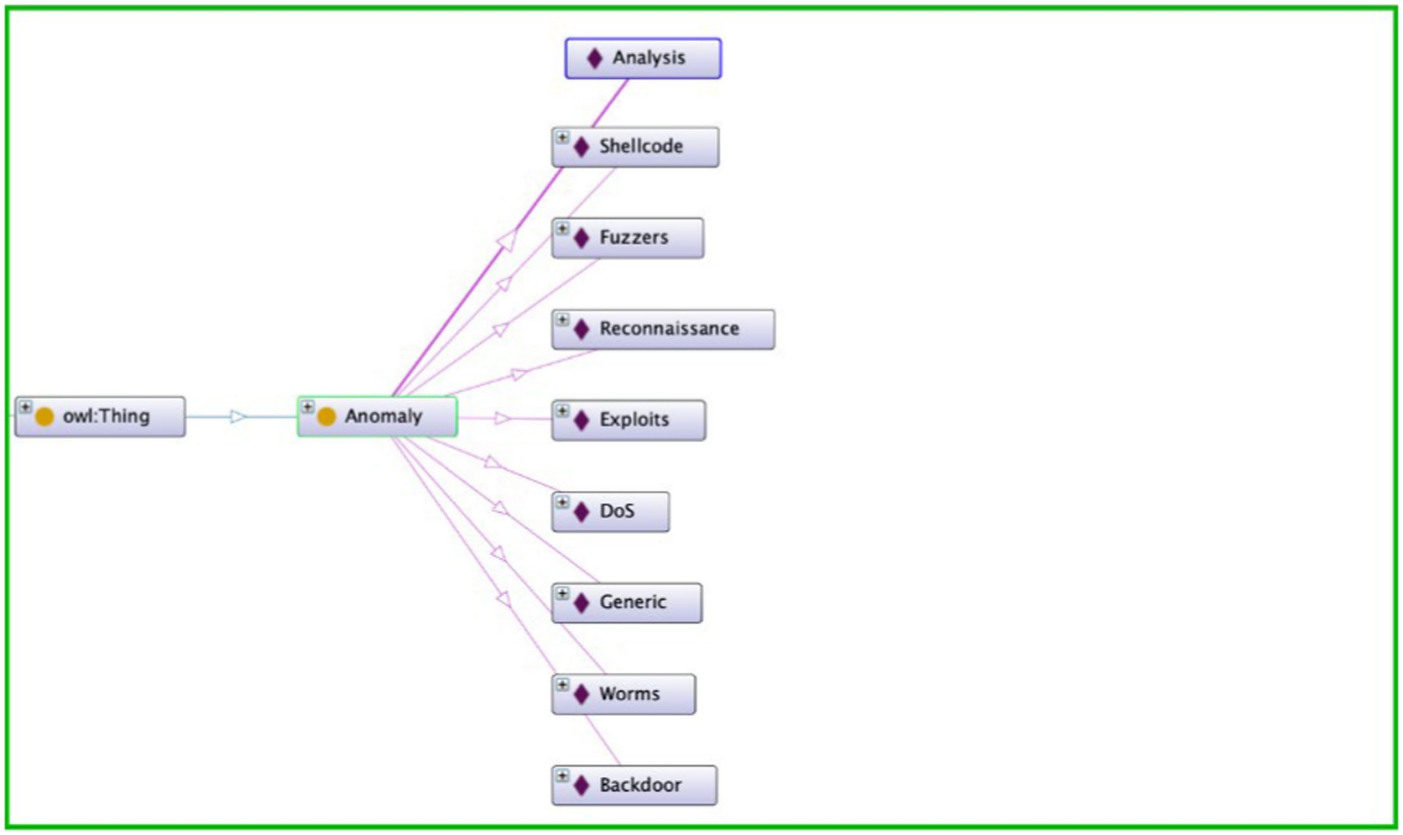
Ontology rule creations.

The semantic layer introduced by the ontology enriches the feature space within the dataset, imparting a deeper understanding of the relationships between various entities. In the realm of cyber threats, nuances in data are often subtle and complex. Semantic correlation, facilitated by the ontology, allows the system to discern patterns and relationships that may not be immediately evident through traditional correlation methods. The semantic layer introduces a level of abstraction that enables the system to recognize the significance of seemingly disparate data points, leading to a more comprehensive and insightful analysis. Figure 3 depicts the Anomaly Hierarchy used in our research. Figure 4 depict the ontology rule creations.

#### 3.6.4 Integration of findings

The comprehensive methodology employed in developing the “Cyber Situational Awareness Intelligent System” comprises several key steps. Beginning with Algorithm Selection, the meticulous choice of the Isolation Forest algorithm stands out for its efficacy in identifying anomalies within high-dimensional datasets, thereby providing a foundation for robust anomaly detection in the dynamic cybersecurity landscape. The subsequent training process involves rigorous training of the Isolation Forest algorithm on preprocessed and semantically enriched datasets. This step optimizes the algorithm’s anomaly detection capabilities, ensuring efficient and precise anomaly isolation in real-time cyber threat scenarios. The integration of Structured Threat Information eXpression (STIX) and Feature Mapping forms the third step. STIX provides a standardized language for expressing detailed threat information, while feature mapping systematically links critical features like src_IP and dest_IP, to STIX indicators. This integration enhances semantic understanding and enriches the dataset with contextual threat information. The fourth step involves ontology creation to establish a structured representation of knowledge in the cybersecurity domain. This foundational structure enables the systematic categorization and correlation of different elements, which significantly enhances the system’s ability to contextualize and interpret cyber threat data.

## 4 Results and discussion

### 4.1 Experimental setup

The experiments were conducted on a computer equipped with the following specifications: Intel Xeon Gold 6,230 (20 cores, 40 threads), Base Clock Speed: 2.1 GHz, 27.5 MB (Cache), and 64 GB RAM (Memory). The python programing language and TensorFlow framework are used to develop the system.

### 4.2 Dataset descriptions

The UNSW-NB15 (Zoghi and Serpen, 2024; Alsharaiah et al., 2024; Stiawan et al., 2020; Online Repository, 2021) dataset is a widely used benchmark dataset in the field of cybersecurity and network intrusion detection. It was created by the University of New South Wales (UNSW) in Australia and consists of network traffic data collected in a controlled environment for the purpose of research and analysis in the domain of network security. The UNSW-NB15 dataset is of moderate size, containing tens of thousands of network flow records. This size allows for meaningful analysis and experimentation while also being manageable for processing and storage. The dataset exhibits class imbalance, with the majority of records representing normal traffic and a smaller proportion representing attack instances. This reflects the typical imbalance between normal traffic and malicious activities in real-world network environments.

### 4.3 Feature engineering

A thorough feature engineering procedure is crucial for mining valuable insights from datasets. In our analytical preparation, we carried out an extensive feature engineering process, introducing key features like src_IP and dest_IP to apprehension vital details about source and destination IP addresses. This collection was strategically made to enhance semantic understanding by aligning with STIX indicators. By integrating src_IP and dest_IP, our approach forges a critical link between the dataset and STIX indicators, leading to a deeper semantic interpretation of network traffic.

### 4.4 Evaluation matrices

The performance of the proposed system was evaluated in comparison with the existing system based on accuracy, recall, precession, F1 score, and error rate. Accuracy paints a broad picture of overall correctness, while the F1 Score delves deeper by harmonizing precision and recall, balancing the trade-off between finding true positives and avoiding false ones. The error rate serves as a complementary angle, highlighting the percentage of errors across both false positives and negatives. For situations prioritizing the capture of relevant instances, Recall reveals the success rate of identifying true positives. In contrast, precision sheds light on the accuracy of positive predictions, minimizing false positives that may be crucial depending on the given task. The evaluation metrics are defined using Equations 7–11


(7) $$\begin{array}{l} \mathrm{Accuracy}=\\ \left(\mathrm{True}\;\mathrm{Positives}+\mathrm{True}\;\mathrm{Negatives}\right)/\left(\mathrm{Total}\;\mathrm{Predictions}\right) \end{array}$$

(8) $$F1\;\mathrm{Score}=2*\left(\mathrm{Precision}*\mathrm{Recall}\right)/\left(\mathrm{Precision}+\mathrm{Recall}\right)$$

(9) $$\begin{array}{l} \mathrm{Error}\;\mathrm{Rate}=\\ \left(\mathrm{False}\;\mathrm{Positives}+\mathrm{False}\;\mathrm{Negatives}\right)/\left(\mathrm{Total}\;\mathrm{Predictions}\right)\; \end{array}$$

(10) $$\mathrm{Recall}=\mathrm{True}\;\mathrm{Positives}/\left(\mathrm{True}\;\mathrm{Positives}+\mathrm{False}\;\mathrm{Negatives}\right)$$

(11) $$\begin{array}{l} \mathrm{Precision}=\\ \mathrm{True}\;\mathrm{Positives}/\left(\mathrm{True}\;\mathrm{Positives}+\mathrm{False}\;\mathrm{Positives}\right) \end{array}$$

### 4.5 Contextual detection rate

The Contextual Detection Rate is a tailored metric designed to account for the specific nuances of a given environment or situation, especially in scenarios where different types of threats have varying levels of importance or severity. This metric goes beyond the traditional detection rate (or recall) by incorporating context-specific factors that reflect the priorities or risks associated with different threats. Contextual detection rate is computed using the Equation 12.


(12) $$\mathrm{Contextual}\;\mathrm{detection}\;\mathrm{rate}=\frac{\sum \left(c_{i}\times TP_{i}\right)}{(c_{i}\times \left(TP_{i}+FN_{i}\right)}$$

Where $c_{i}$ represents a context-specific factor for each threat type i.

are the true positives and false negatives for each threat type i.

### 4.6 Performances evaluations using UNSW-NB15 dataset

The comparative analysis of unsupervised machine learning algorithms, including our proposed system, Isolation Forest, Local Outlier Factor (LOF), and Autocoder, was conducted on the UNSW-NB15 dataset. The dataset was divided into 80% for model training and 10% for 10-fold cross-validation to adjust the hyperparameters and evaluate generalizability. Finally, the remaining 10% served as an independent test set for an unbiased evaluation of the model’s final performance.

**Figure 5:**
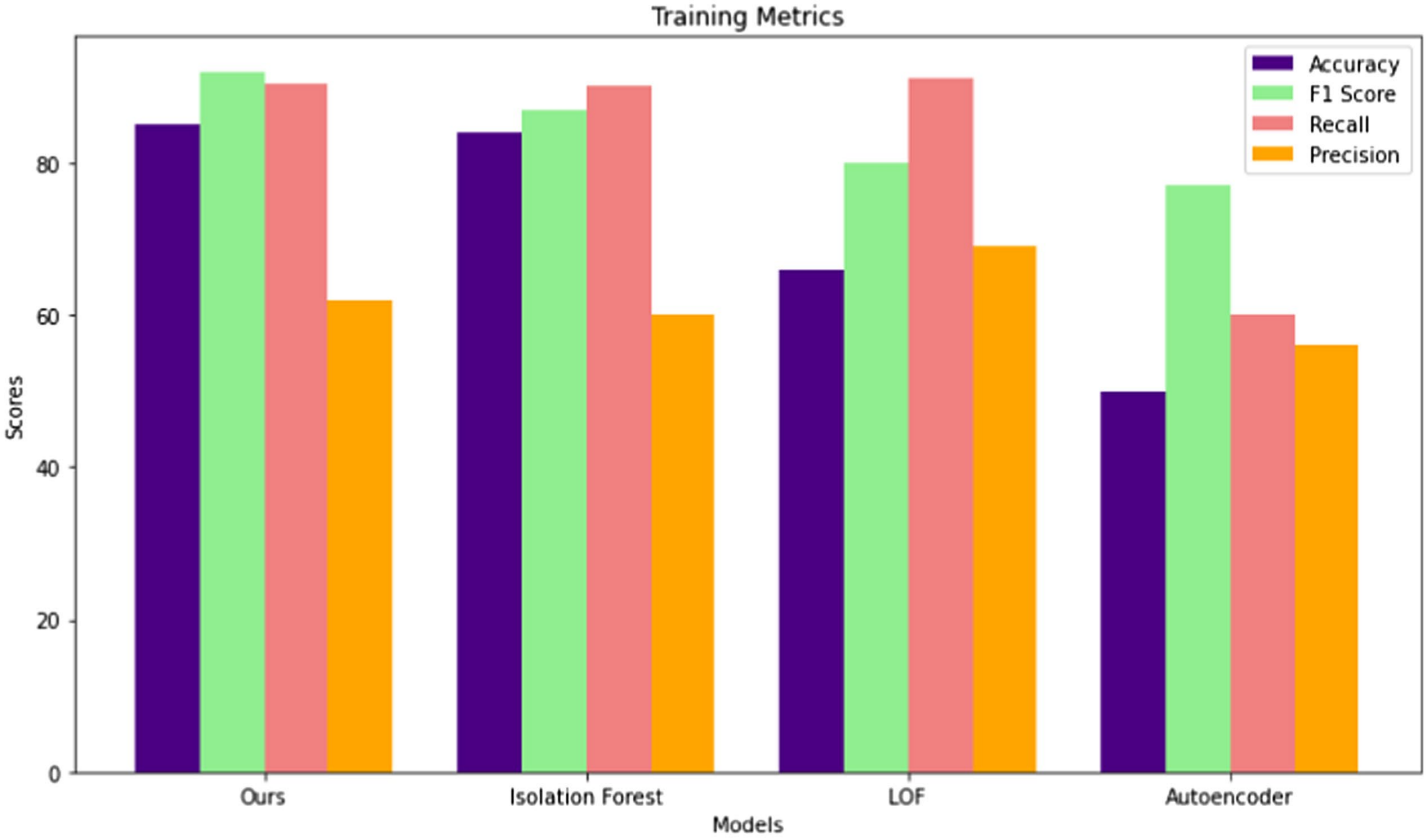
Comparison of model performance during the training phase across different models.

In the training phase, four anomaly detection models were evaluated across four key metrics of Accuracy, F1 Score, Recall, and Precision. Among these models, the proposed model demonstrated the highest performance across all metrics, achieving an accuracy of 85.04%, an F1 Score of 92%, a recall rate of 90.43%, and a precision of 62%. The isolation forest model was closely followed, with an accuracy of 84%, an F1 Score of 87%, a recall rate of 90%, and a precision of 60%. The LOF (Local Outlier Factor) model exhibited slightly lower performance, achieving an accuracy of 66%, an F1 Score of 80%, a recall rate of 91%, and a precision of 69%. The Autoencoder model consistently demonstrated the lowest performance across all metrics, with an accuracy of 50%, an F1 Score of 77.05%, a recall rate of 60%, and a precision of 56%. These results highlight the effectiveness of the proposed and Isolation Forest models in accurately identifying anomalies during the training phase, while also underscoring the limitations of the Autoencoder model in this context. Figure 5 presents performance comparisons of the different models during the training phase.

The performance of four different anomaly detection models, of proposed model, Isolation Forest, LOF, and Autoencoder, was evaluated across four key metrics: Accuracy, F1 Score, Recall, and Precision in the testing phase.

**Figure 6:**
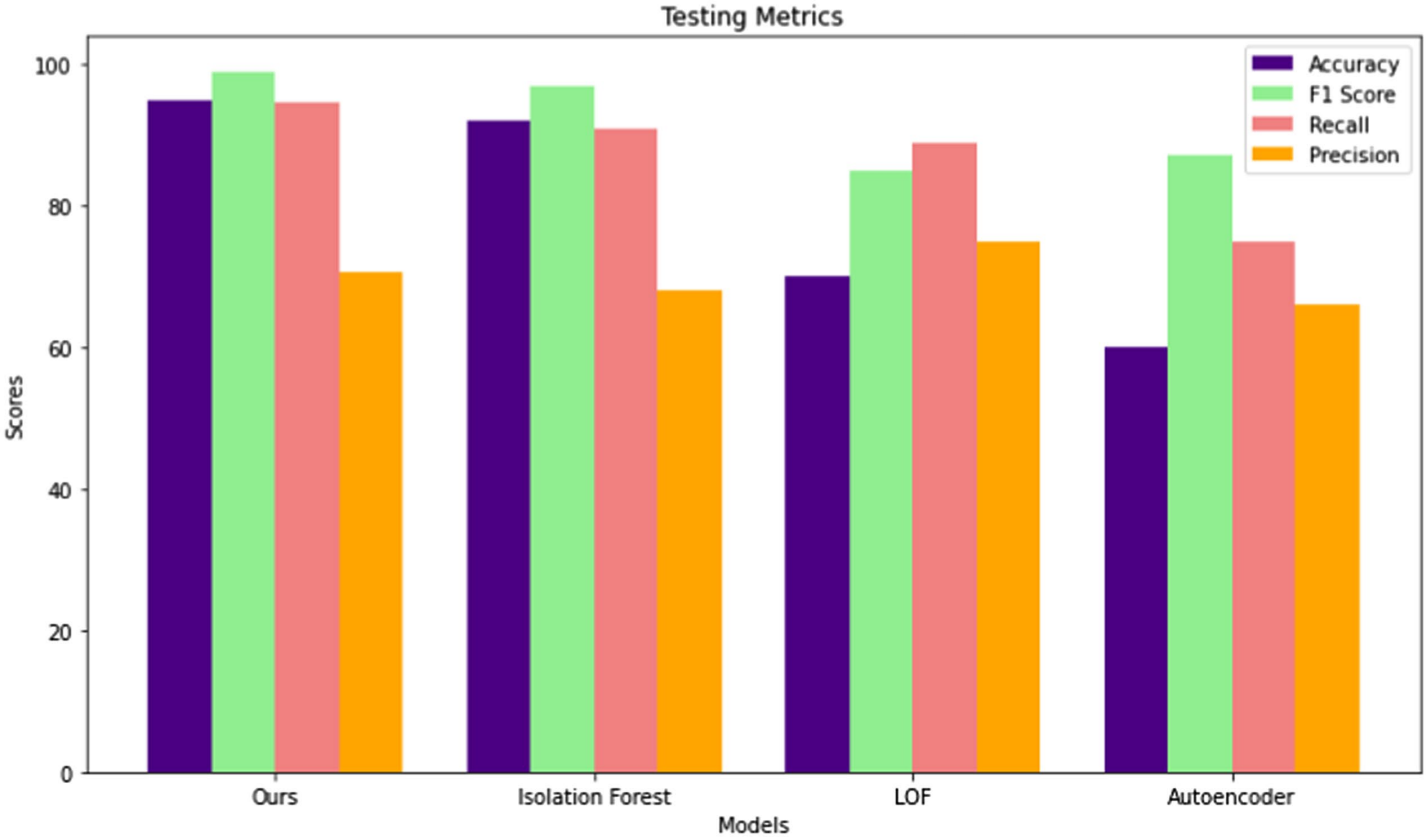
Comparison of model performance during the testing phase across different models.

Among these models, proposed model demonstrated superior performance across all metrics, achieving the highest Accuracy (95%) and F1 Score (99%). It also boasted the highest recall rate (94.60%), indicating its effectiveness in identifying true positives. However, the Autoencoder model consistently exhibited the lowest performance across all metrics, with the lowest Accuracy (60%) and F1 Score (87.24%), indicating its limitations in accurately detecting anomalies. Notably, the LOF model performed well in terms of Precision (75%), although its overall performance was surpassed by both proposed model and Isolation Forest models. These results underscore the importance of comprehensive evaluation and comparative analysis when selecting the most suitable anomaly detection model for a given dataset. Figure 6 shows the performances comparisons in Testing phase for different model.

**Figure 7:**
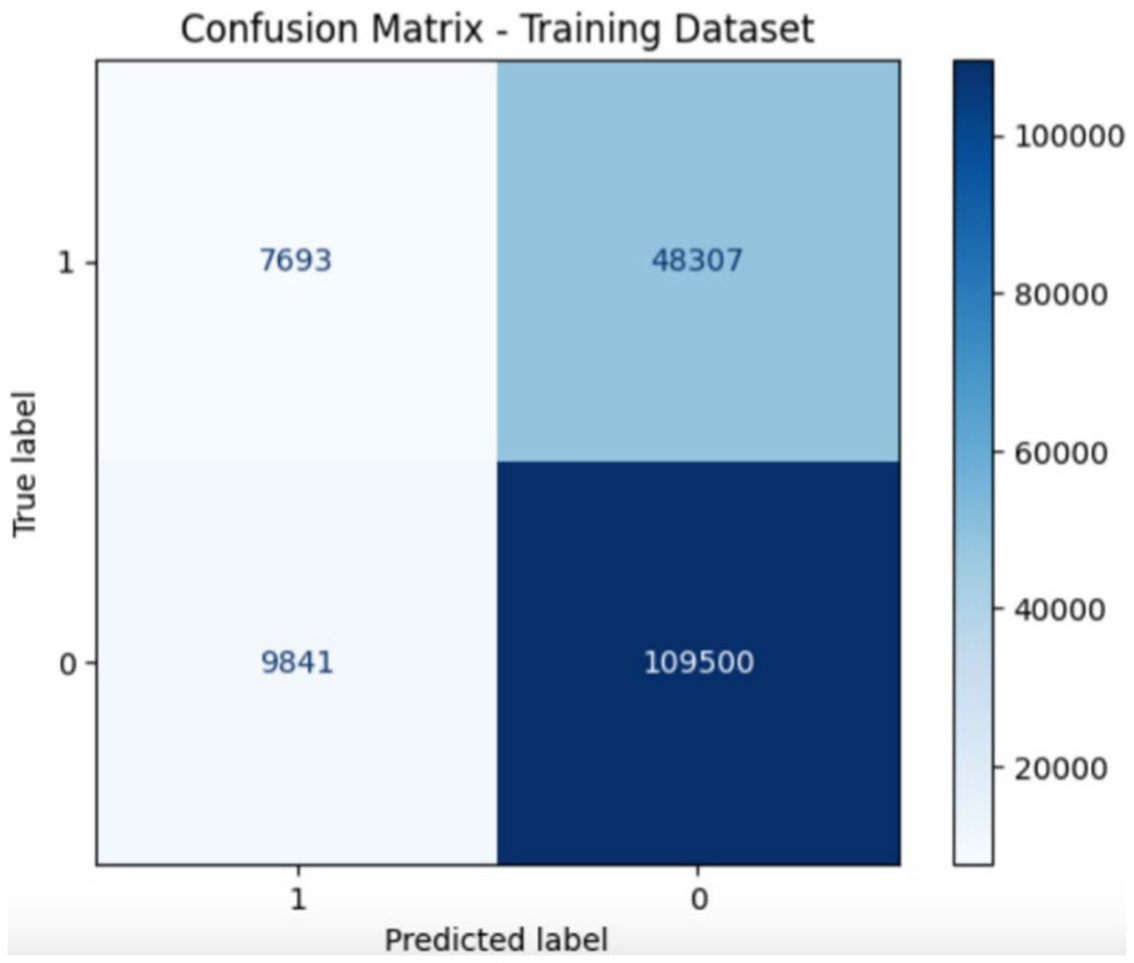
Confusion matrix of the proposed models during training.

**Figure 8:**
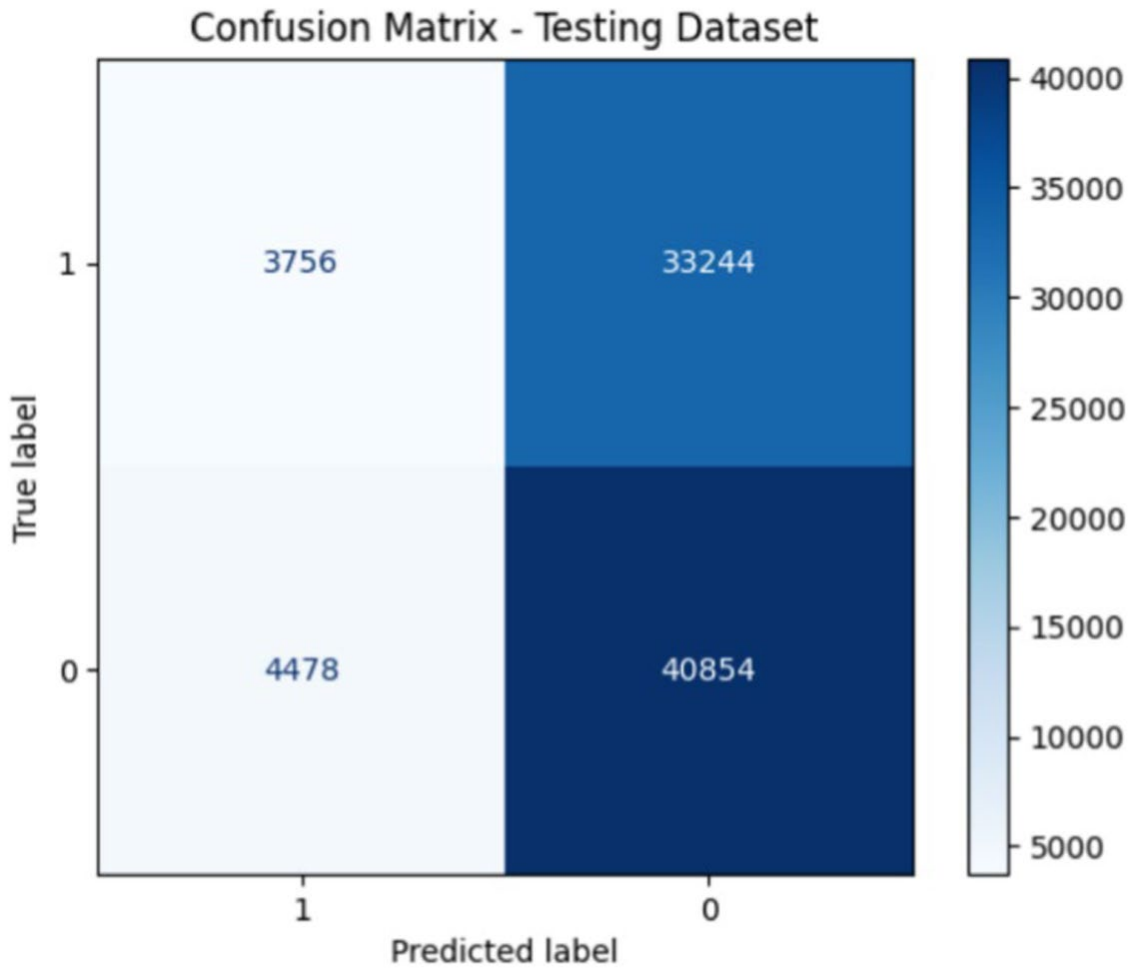
Confusion matrix of the proposed models during testing.

Figures 7, 8 show the confusion matrix of the proposed training and testing. In the confusion matrix for the proposed model using UNSW-NB15 during training, the values were as follows: 7693 True Positives (TP), 4,803 False Positives (FP), 9,841 False Negatives (FN), and 109,500 True Negatives (TN). This indicates that the model correctly identified 7,693 malicious instances and 109,500 benign instances; however, it incorrectly flagged 4,803 benign instances as malicious and missed 9,841 malicious instances. In the confusion matrix for the proposed model during testing on the UNSW-NB15 dataset, the values are: 3754 True Positives (TP), 33,244 False Positives (FP), 4,478 False Negatives (FN), and 40,854 True Negatives (TN). This means that the model correctly identified 3,754 malicious and 40,854 benign traffic instances. However, it incorrectly classified 33,244 benign instances as malicious and missed 4,478 malicious instances. The accuracy of the model is approximately 95%; thus, there is room for improvement in terms of reducing false positives and false negatives to enhance the overall detection effectiveness.

Table 2 compares detection rates for state-of-the-art systems. Among the compared models, our model emerged as the frontrunner in terms of detection rate, boasting an impressive score of 95. A proper ontology was created to address the unique types of threats in the application context. The existing ontologies did not adequately cover the variety and specificity of these threats, particularly in terms of facilitating structured knowledge representation and facilitating automated reasoning for contextual understanding. This proprietary ontology enables a tailored approach for categorizing threat data, aligning with the STIX protocol, and facilitating data interoperability.

**Table 2:** Comparison of detection rates for state-of-the-art models.

Model	Ours	Situational awareness and CPS (Park et al., 2019)	PRAETORIAN (Nota and Petraglia, 2024)	HoneyNet (Friedberg et al., 2015)
Detections rate	95	93	92	89

**Figure 9:**
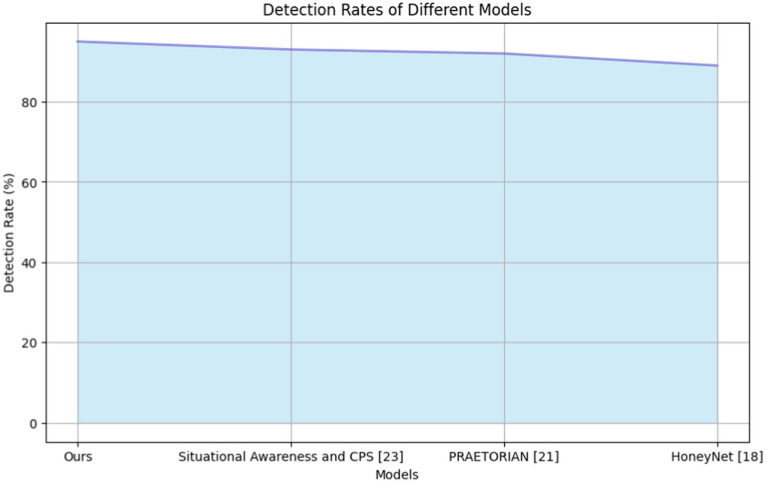
Detection rate comparisons for state-of-the art system.

This indicates the robustness of detecting and identifying potential cybersecurity threats in the system. The next is situational awareness and cyber-physical systems (Park et al., 2019), with a commendable detection rate of 93, demonstrating solid performance in situational awareness within cyber-physical systems. The PRAETORIAN (Papadopoulos et al., 2024) model provides a detection rate of 92, demonstrating effective detection and analysis capabilities. However, the HoneyNet (Tan et al., 2021) methods have a detection rate score of 89, which is short compared to other models in accurately identifying cybersecurity threats. Figure 9 compares the detection rates of the state-of-the-art systems.

### 4.7 Performance evaluation on the CICIDS 2017 dataset

The performances of various state-of-the-art systems on the CICIDS 2017 dataset (Stiawan et al., 2020; Online Repository, 2017) is compared based on several metrics, including accuracy, F1 Score, Error Rate, Recall, and Precision. The proposed method achieved the highest Accuracy of 97% and an impressive F1 Score of 0.945. These metrics indicate robust performance in accurately identifying normal and anomalous network activities. With an Error Rate of 3.2%, the proposed system demonstrated a low rate of misclassifications, highlighting its reliability in distinguishing between benign and malicious network behaviors. High Recall (0.96) and Precision (0.93) values further underscore the effectiveness of the proposed model in capturing true positive instances while minimizing false alarms, which is crucial for maintaining high detection rates without triggering unnecessary alerts. Table 3 compares the performances of the state-of-the-art systems on the CICIDS 2017 Dataset.

**Table 3:** Performance comparisons of state-of-the-art systems on the CICIDS 2017 dataset.

Method	Accuracy	F1 Score	Error Rate (%)	Recall	Precision
Ours	97	0.945	3	0.96	0.93
Situational Awareness and CPS (Park et al., 2019)	89	0.94	11	0.94	0.95
PRAETORIAN (Papadopoulos et al., 2024)	93.2	0.925	6.8	0.93	0.92
HoneyNet (Tan et al., 2021)	92.3	0.91	7.7	0.91	0.92

Situational Awareness and CPS (Park et al., 2019) achieved an accuracy of 89% and an F1 Score of 0.94, which indicates good overall performance in classification tasks. The proposed method demonstrated a relatively low Error Rate of 11%, which demonstrates its reliability in practical deployment scenarios. High Recall (0.94) and Precision (0.95) values suggest strong performance in correctly identifying malicious activities while maintaining a high level of precision. These attributes make it suitable for applications in which minimizing false positives is critical.

PRAETORIAN (Papadopoulos et al., 2024) achieved an accuracy of 93.2% and an F1 Score of 0.925, which indicates robust performance in accurately classifying network traffic. Despite a slightly higher Error Rate of 6.8%, PRAETORIAN (Papadopoulos et al., 2024) maintains strong overall performance with high Recall (0.93) and Precision (0.92) values, effectively detecting intrusions while balancing false positive rates. These results highlight the effectiveness of the proposed method in real-world scenarios where comprehensive threat detection is paramount.

Honeynet (Tan et al., 2021) achieved an accuracy of 92.3% and an F1 Score of 0.91, demonstrating reliable performance in classifying network activities. However, with an Error Rate of 7.7%, Honeynet (Tan et al., 2021) demonstrated a higher misclassification rate compared to other methods, potentially affecting its suitability in high-stake cybersecurity environments. The balanced recall (0.91) and Precision (0.92) values indicate effective detection capabilities, although the false positive rate is slightly higher than that of the top-performing methods. These findings underscore the importance of evaluating intrusion detection systems based on multiple metrics to assess their suitability to various cybersecurity challenges.

The proposed model outperformed other state-of-the-art methods on the CICIDS 2017 dataset in terms of Accuracy, F1 Score, and Error Rate. It achieves a high level of precision (0.93) and recall (0.96), and it demonstrates superior capability in identifying and classifying both normal and anomalous network behaviours. Situational Awareness and CPS (Javadnejad et al., 2024), PRAETORIAN (Papadopoulos et al., 2024), and Honeynet (Tan et al., 2021) also exhibit strong performances with varying strengths in accuracy and error rates. These findings highlight the importance of robust evaluation metrics when assessing the effectiveness of intrusion detection systems for cybersecurity applications.

## 5 Conclusion

The proposed method enhances cyber situational awareness by applying advanced algorithms, standardized threat information expression, and structured knowledge representation. The Isolation Forest algorithm, in particular, emerges as a standout anomaly detection algorithm supported by rigorous training and optimization processes. The integration of STIX and Feature Mapping enriches datasets with contextual threat information, and ontology development facilitates semantic correlation and dynamic assessment of threat intelligence data. The experimental results demonstrate the effectiveness of the proposed methodology. The proposed model consistently outperformed the alternatives in terms of accuracy, precision, recall, and error rate. Overall, the results of this study contribute to the evolution of proactive cybersecurity strategies that foster resilience and adaptability when addressing emerging cyber threats. Existing methods in the intelligence information and ontology systems field have performance and scalability limitations. A lack of information security knowledge can lead to inadequate risk management strategies, which highlights the need for comprehensive ontologies that cover various aspects of cybersecurity.

### 5.1 Future work

In future, we plan to improve existing intelligence systems and ontologies to handle more cybersecurity aspects effectively. This involves developing more advanced ontologies to better cover emerging threats. In addition, exploring new deep learning methods like generative Adversarial network can enhance how accurately we detect anomalies. It is also important to expand the use of frameworks like STIX for real-time data fusion and automated decision-making, which are crucial for quickly responding to threats. Finally, conducting long-term studies in real-world settings will validate and improve our methodology’s effectiveness in dynamic cyber environments.

## Data Availability

The original contributions presented in the study are included in the article/supplementary material, further inquiries can be directed to the corresponding author/s.
